# A randomized controlled trial of iliopsoas plane block vs. femoral nerve block for hip arthroplasty

**DOI:** 10.1186/s12871-023-02162-5

**Published:** 2023-06-08

**Authors:** Chun-guang Wang, Zhi-qiang Zhang, Yang Yang, Yu-bin Long, Xiu-li Wang, Yan-ling Ding

**Affiliations:** 1 Department of Anesthesiology, The First Central Hospital of Baoding, Northern Great Wall Street 320#, Baoding, 071000 Hebei China; 2 Department of Cardio-Thoracic Surgery, The First Central Hospital of Baoding, Baoding, 071000 China; 3 Department of Orthopedics, The First Central Hospital of Baoding, Baoding, 071000 Hebei China; 4 Department of Anesthesiology, The Third Hospital of Hebei Medical University, Shijiazhuang, 050051 China

**Keywords:** Hip arthroplasty, Iliopsoas plane block, Femoral nerve block

## Abstract

**Background:**

Iliopsoas plane block (IPB) is a novel analgesic technique for hip surgery that retains quadriceps strength. However, evidence from randomized controlled trial is remains unavailable. We hypothesized that IPB, as a motor-sparing analgesic technique, could match the femoral nerve block (FNB) in pain management and morphine consumption, providing an advantage for earlier functional training in patients underwent hip arthroplasty.

**Methods:**

We recruited ninety patients with femoral neck fracture, femoral head necrosis or hip osteoarthritis who were scheduled for unilateral primary hip arthroplasty were recruited and received either IPB or FNB. Primary outcome was the pain score during hip flexion at 4 h after surgery. Secondary outcomes included quadriceps strength and pain scores upon arrival at post anesthesia care unit (PACU) and at 2, 4, 6, 24, 48 h after surgery, the first time out of bed, total opioids consumption, patient satisfaction, and complications.

**Results:**

There was no significant difference in terms of pain score during hip flexion at 4 h after surgery between the IPB group and FNB group. The quadriceps strength of patients receiving IPB was superior to those receiving FNB upon arrival at PACU and at 2, 4, 6 and 24 h after surgery. The IPB group showed a shorter first time out of bed compared to the FNB group. However, there were no significant differences in terms of pain scores within 48 h after surgery, total opioids consumption, patient satisfaction and complications between the two groups.

**Conclusion:**

IPB was not superior to FNB in terms of postoperative analgesia for hip arthroplasty. However, IPB could serve as an effective motor-sparing analgesic technique for hip arthroplasty, which would facilitate early recovery and rehabilitation. This makes IPB worth considering as an alternative to FNB.

**Trial registration:**

The trial was registered prior to patient enrollment at the Chinese Clinical Trial Registry (ChiCTR2200055493; registration date: January 10, 2022; enrollment date: January 18, 2022; https://www.chictr.org.cn/searchprojEN.html).

## Introduction

Hip arthroplasty is recognized as an effective therapy for end-stage osteoarthritis of the hip, femoral neck fracture, and femoral head necrosis. However, patients who underwent hip arthroplasty often experience moderate to severe postoperative pain, which hampers early mobilization, prolongs hospital stay, and worsens postoperative function. Opioids, the mainstay of postoperative pain control, are associated several undesirable side effects such as dizziness, sedation, nausea, and vomiting [1]. With the advent of ultrasound technology, peripheral nerve block is increasingly used for postoperative analgesia in patients undergoing hip arthroplasty [2–4]. Lumbar plexus block, quadratus lumborum block, fascia iliaca compartment block and femoral nerve block (FNB) can provide reliable analgesia and reduce opioid consumption for hip arthroplasty [5–14]. However, all these methods can weaken quadriceps muscle strength, hampering early mobilization and increasing the risk of falls.

Optimal regional analgesia for hip arthroplasty should expedite recovery and rehabilitation. This requires not only minimizing postoperative pain during activity but also maximizing the retention of mobility. The pericapsular nerve group (PENG) block, which has been successfully employed for analgesia in patients with hip fracture and surgery, and has been shown to facilitate early postoperative mobilization [15–18]. However, some recent studies have reported quadriceps motor block following PENG block [19–21]. The iliopsoas plane block (IPB) is a novel motor-sparing technique that selectively targets the sensory branches of the hip joint originating from femoral nerve and accessory obturator nerve [22–24]. Our recent studies suggested that IPB could provide effective analgesia for patients underwent hip surgery, while preserving quadriceps strength [25–27]. However, evidence from randomized controlled trials remains unavailable. We hypothesized that IPB, as a motor-sparing analgesic technique, could match FNB in terms of pain management and morphine consumption, and provide advantage for earlier functional training. In this randomized controlled trial, we compared the effects of IPB and FNB in patients undergoing hip arthroplasty.

## Materials and methods

This randomized controlled trial received approval from the Medical Ethics Committee of the First Central Hospital of Baoding ([2021] 181), and written informed consent was obtained from all subjects. The trial was registered prior to patient enrollment at Chinese Clinical Trial Registry (ChiCTR2200055493; registration date: January 10, 2022; enrollment date: January 18, 2022; https://www.chictr.org.cn/searchprojEN.html). Ninety patients scheduled for unilateral primary posterior approach hip arthroplasty were recruited. The inclusion criteria were: age between 18 and 80 years, American Society of anesthesiologists (ASA) physical status I to III, and patient with femoral neck fracture or femoral head necrosis or hip osteoarthritis. The exclusion criteria included: chronic kidney disease or cardiac insufficiency, chronic use of analgesics or psychotropics, allergy to ropivacaine, contraindication to nerve block, limb neuropathy on the operative side, and inability to comprehend or cooperate to accomplish this study.

Patients were randomly assigned to either the IPB group or the FNB group in a 1:1 ratio, based on a computer-generated randomization sequence. Random allocation was executed using a sealed envelope containing a numbered card, which was not opened until the nerve block was implemented. Except for the nerve block team, which included a senior anesthesiologist and an anesthesia nurse, all other participants (junior anesthesiologists participating in assessment, nurses on the floor, surgeons and patients) were blinded to the randomization.

### Nerve block guided by ultrasound

**Fig. 1:**
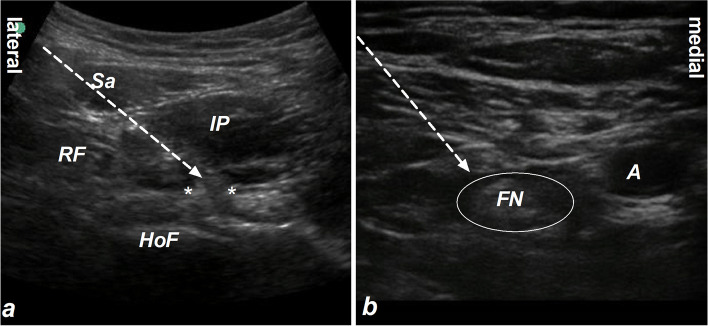
Iliopsoas Plane Block (**a**) and Femoral Nerve Block (**b**). Sa represents the Sartorius muscle, RF represents the Rectus Femoris muscle, IP represents the Iliopsoas muscles, and HoF represents the Head of Femur. The white asterisk indicates the Iliofemoral ligament, FN represents the Femoral Nerve, “A” represents the Femoral Artery, and the white arrow shows the needle trajectory of the nerve block

In the IPB group, IPB was performed under ultrasound guidance as previous reports (Fig. 1a) [23]. With a supine position, a low-frequency ultrasound probe (M-Turbo, Sonosite, USA) was placed distal to anterior superior iliac spine in the transverse plane. And then, the probe was rotated about 30 degrees in an anticlockwise direction, and slid along the inguinal ligament until the head of femur entering the acetabular rim. After local infiltration of 1% lidocaine, a needle was penetrated through the sartorius and iliopsoas muscle and reached into the iliopsoas plane between the iliopsoas muscle and the iliofemoral ligament. Once the needle tip’s position was confirmed, 10 ml of 0.5% ropivacaine was injected.

For patients in the FNB group, FNB was performed under ultrasound guidance (Fig. 1b) [13]. With a supine position, a high-frequency linear transducer (M-Turbo, Sonosite, USA) was placed on the inguinal crease. The femoral artery and femoral nerve were identified clearly. After local infiltration of 1% lidocaine, a needle was inserted using an in-plane approach from lateral to medial direction. Once the needle tip’s position was confirmed, 10 ml of 0.5% ropivacaine was injected adjacent to the femoral nerve.

Lateral femoral cutaneous nerve block was performed with 5 ml of 0.5% ropivacaine for patients in both the IPB and FNB group.

### General anesthesia and postoperative analgesia

All nerve block procedures were performed by the same senior anesthesiologist after anesthesia induction. For the induction of anesthesia, midazolam 0.05 mg/kg, etomidate 0.1 ~ 0.2 mg/kg, rocuronium 0.6 mg/kg, and remifentanil 1 ~ 2 μg/kg were administered intravenously. Endotracheal intubation was inserted after muscle relaxation. The maintenance of anesthesia was achieved with sevoflurane at a minimum alveolar concentration of 0.8 to 1, remifentanil at 0.1 to 0.3 μg/kg/min, propofol 2 ~ 5 mg/kg/h and intermittent doses of rocuronium. Throughout the operation, ETCO2 was maintained at 35 to 40 mmHg, BIS was kept at 45 to 55, and the fluctuation of MAP and HR did not exceed ± 10% of the baseline. All hip arthroplasty procedures were performed by the same surgeon. After the operation, patients received an intravenous injection of flurbiprofen 50 mg, administrated twice a day.

### Outcomes

The primary outcome was the pain score during hip flexion at 4 h post-surgery. Secondary outcomes included the quadriceps strength and pain scores upon arrival at the post-anesthesia care unit (PACU) and at 2, 4, 6, 24, 48 h post-surgery, the first time out of bed, total opioids consumption, patient satisfaction, and complications. Postoperative pain was evaluated using the visual analog scale (VAS) (0–10; 0: no pain, 10: worst pain). Rescue analgesia with opioids was administered when the VAS score exceeded 3. The quadriceps strength was assessed using the manual muscle testing (MMT) grade (0–5; 0: no muscle contraction, 1: muscle contraction present but unable move joint, 2: able to move joint but not resist gravity, 3: able to resist gravity but not bear substantial resistance, 4: able to resist some level of substantial resistance, 5: able to resist full resistance).

### Statistical analysis

According to Lin’s Report, the mean pain score could be reduced 3.4 points using FNB with an SD of 2 points [19]. Our experience indicated that the mean pain score can be reduced 6 points by IPB, with an SD of 3 points. A sample size of 38 patients was required to achieve 95% power to detect a difference at a 5% level of a two-tailed type I error. To account for potential dropouts or protocol violations, a total of 90 subjects were enrolled in this study.

Statistical analyses were performed using SPSS 16.0 software. The Kolmogorov–Smirnov test was used to check for normal distribution of data. Body mass index was expressed as mean (standard deviation) and compared using a *t*-test. Variables such as age, surgery duration, pain score, quadriceps strength, opioid consumption, the first time out of bed, and patient satisfaction were expressed as median (IQR [range]) and compared using the Mann–Whitney *U* test. Categorical data such as gender, ASA physical status, type of hip pathology, type of surgery and complications were expressed as number (%) and compared using the *Pearson Chi-Square Test* or *Fisher’s Exact Test*. *P* < *0.05* was considered statistically significant.

## Results

**Fig. 2:**
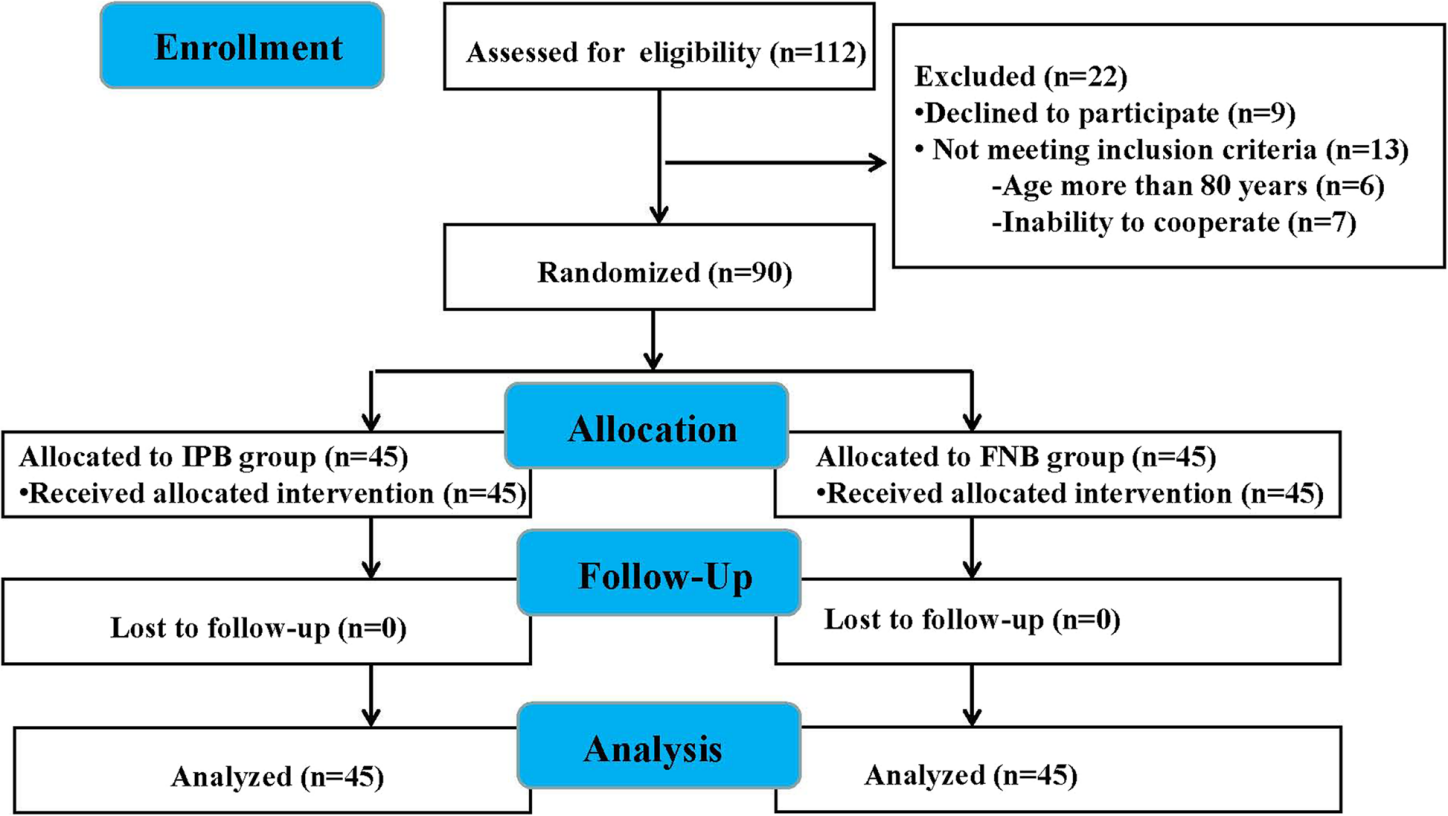
Study Flow Diagram. IPB represents the Iliopsoas Plane Block, and FNB represents the Femoral Nerve Block. This diagram provides a detailed representation of the number of patients initially recruited, those excluded, and those ultimately included in the study

One hundred and twelve patients were initially recruited. Nine patients declined to participate, six patients were older than 80 years, and seven patients were unable to cooperate with the assessment. Thus 90 patients were ultimately enrolled and completed the study (Fig. 2). There were no significant differences in patient characteristics and surgery duration between the IPB group and FNB group (Table 1).

**Table 1:** Patient and perioperative characteristics. Values are median (IQR [range]), number (proportion) or mean (SD)

	**IPB (*n* = 45)**	**FNB (*n* = 45)**
**Age; y**	64 (56.5–70 [38–80])	65 (57.5–70.5 [31–80])
**Gender**		
Male	25 (56%)	25 (56%)
Female	20 (44%)	20 (44%)
**ASA**		
I	16 (36%)	13 (29%)
II	24 (53%)	26 (58%)
III	5 (11%)	6 (13%)
**BMI**; kg.m^−2^	25.58(3.68)	24.78(3.88)
**Type of Hip Pathology**		
Femoral neck fracture	20 (44%)	20 (44%)
Femoral head necrosis	25 (56%)	21 (47%)
Hip osteoarthritis	0 (0%)	4 (9%)
**Type of Surgery**		
Total hip arthroplasty	39 (87%)	41 (91%)
Hip hemiarthroplasty	6 (13%)	4 (9%)
**Duration of surgery**; min	110 (88.5–129 [56–180])	105 (88–133 [60–203])

*IPB* Iliopsoas plane block, *FNB* Femoral nerve block, *ASA* American Society of anesthesiologists, *BMI* Body mass index

No significant differences were observed between the IPB group and FNB group in terms of pain score during hip flexion at 4 h after surgery (*P* > *0.05*, Table 2). Similarly, no significant differences were found in terms of pain scores at rest upon arrival at PACU, at 2, 4, 6, 24 and 48 h after surgery between the two groups (*P* > *0.05*, Table 2). There were also no significant differences in terms of pain scores during flexion of hip upon arrival at PACU, at 2, 6, 24, and 48 after surgery between the two groups (*P* > *0.05*, Table 2).

**Table 2:** Outcomes. Values are median (IQR [range]) or number (proportion)

	IPB (*n* = 45)	FNB (*n* = 45)	*P* value
***Primary Outcome***			
Pain Score during flexion of hip at 4 h	1 (0–2[0–4])	1 (1–2[0–5])	0.896
***Secondary Outcome***			
Pain score at rest in PACU	0 (0–1[0–4])	0 (0–0.5[0–4])	0.491
Pain score at rest at 2 h	0 (0–1[0–4])	0 (0–1[0–2])	0.988
Pain score at rest at 4 h	0 (0–1[0–3])	0 (0–1[0–3])	0.849
Pain score at rest at 6 h	0 (0–0.5[0–3])	0 (0–1[0–3])	0.554
Pain score at rest at 24 h	0 (0–0 [0–3])	0 (0–0[0–2])	0.542
Pain score at rest at 48 h	0 (0–0 [0–4])	0 (0–0[0–1])	0.887
Pain Score during flexion of hip in PACU	1 (0–2[0–5])	1 (0–1[0–6])	0.862
Pain Score during flexion of hip at 2 h	1 (0–2[0–5])	1 (1–2[0–4])	0.713
Pain Score during flexion of hip at 6 h	1 (0–2[0–4])	1 (1–1.5[0–4])	0.469
Pain Score during flexion of hip at 24 h	1 (1–2[0–4])	1 (1–1[0–2])	0.080
Pain Score during flexion of hip at 48 h	1 (0–2[0–5])	1 (1–1 [0–3])	0.418
Quadriceps strength in PACU	4 (3–4[2-5])	1 (1–2[1,2])	0.000
Quadriceps strength at 2 h	4 (4–5[3-5])	1 (1–2[1,2,3])	0.000
Quadriceps strength at 4 h	5 (4–5[3-5])	2 (1–2.5[1-4])	0.000
Quadriceps strength at 6 h	5 (5–5[4,5])	3 (2–3[2-5])	0.000
Quadriceps strength at 24 h	5 (5–5[4,5])	4 (3–5[3,4,5])	0.000
Quadriceps strength at 48 h	5 (5–5[4,5])	5 (5–5[3,4,5])	0.089
Total opioids consumption; mg OME	30 (0–45 [0–60])	30 (0–45[0–90])	0.772
First time out of bed; h	8 (6.5–11[4-14])	24(20–28[18–33])	0.000
Patient satisfaction	9 (8–10[5-10])	9 (8–10[6-10])	0.20
Dizziness	2 (4%)	3 (7%)	1.000
Nausea and vomiting	5 (11%)	5 (11%)	1.000
Nerve injury	0 (0%)	0 (0%)	-
Vascular injury	0 (0%)	0 (0%)	-
Infection	0 (0%)	0 (0%)	-
Delirium	0 (0%)	1 (2%)	1.000
Deep venous thrombosis	3 (7%)	2 (4%)	1.000
Falls in-hospital	0 (0%)	1 (2%)	1.000

*IPB* Iliopsoas plane block, *FNB* Femoral nerve block, *VAS* Visual analog scale, *MMT* Manual muscle testing, *OME* Oral morphine equivalent

However, the quadriceps strength was better in the IPB group than the FNB group upon arrival at PACU, at 2, 4, 6, and 24 h after surgery (*P* < *0.05*, Table 2). Additionally, patients in the IPB group showed a sooner first time out of bed than those in the FNB group, (*P* < *0.05*, Table 2).

There were no significant differences were found between the IPB group and the FNB group in terms of opioids consumption, patient satisfaction and complications (*P* > *0.05*, Table 2).

## Discussion

IPB is a promising motor-sparing analgesic technique that selectively targets the sensory branches of the hip joint originating from femoral nerve and accessory obturator nerve [22, 23]. So far, the analgesic effect of IPB for hip fracture and hip arthroplasty has only been reported in two cases [25, 26]. This randomized controlled trial showed that while IPB was not superior to FNB in term of pain management and morphine consumption. It did prove beneficial for early physical therapy and recovery due to its motor-sparing properties.

The basis for postoperative analgesia for hip arthroplasty lies in the innervation of hip joint. Most nociceptors of the hip joint are situated in the anterior capsule, suggesting that this is the primary site for postoperative analgesia [28]. The femoral nerve, accessory obturator nerve and obturator nerve collectively dominate the sensation of the anterior capsule of the hip joint [29, 30]. However, recent study has shown that postoperative pain relief could not be achieved by obturator nerve block [31], leading us to believe that the principal analgesic targets for the hip joint are the femoral nerve and accessory obturator nerve [22]. In this study, IPB, which selectively targets these sensory branches, demonstrated comparable results to FNB in pain management and morphine consumption, suggesting IPB could provide reliable analgesic effect for patients undergoing hip arthroplasty. Interestingly, there were no differences in pain scores and total opioids consumption between the IPB group and the FNB group. The similar opioids consumption between the two groups may be attributable to the equal degree of postoperative pain, their low baseline opioid consumption and the criteria of our institution to prescribe opioids base on its adverse reaction in elderly. The morphine consumption was lower than that reported in a study by Biboulet P et al., where patients who received FNB consumed less than 0.5 mg/h after 4 h post-surgery [32]. The discrepancy of opioids consumption may be due to the differences in postoperative pain and the criteria for prescribing opioids in different institutions.

Optimal regional analgesia following hip arthroplasty should not only minimize postoperative pain but also maximize motion retention to accelerate recovery and rehabilitation. In this study, the IPB group demonstrated better quadriceps strength retention over the first 24 h after surgery. Greater quadriceps strength facilitates early functional rehabilitation training, leading to fewer complications and quicher recovery. Our findings indicated that the first time out of bed was shortened in the IPB group, likely due to better retention of quadriceps strength. However, it’s important to note that IPB did not entirely avoid the motor block of the quadriceps. For instance, upon arrival at PACU, the quadriceps strength of some patients was no more than an MMT grade 4 out of 5. By 6 h after surgery, all patients in the IPB group demonstrated quadriceps strength was ≥ MMT grade 4 out of 5, sufficient for functional exercise out of bed. We speculate that the initial decline of quadriceps strength might be due to residual anesthetic effect upon arrival at PACU. In contrast, patients in the FNB group showed persistent quadriceps motor block at 6 h after surgery. Most of these patients had quadriceps strength of MMT grade 2 to 3 out of 5, potentially limiting functional exercise out of bed. A recent study found that IPB with 1.8% lidocaine (5 ml) did not significantly reduce the maximal force of knee extension [23]. The unexpected impairment in quadriceps function observed in our study might be due to the high volume of ropivacaine (10 ml) used for IPB. The increased volume might cause an expanded spread of ropivacaine along the articular branches to the trunk of femoral nerve, resulting in motor block [24]. Future studies should aim to determine the optimal volume of local anesthetics for IPB.

There were no nerve or vascular injuries and infections related to nerve block observed in this study. Similarly, there were no differences between the two groups in terms of dizziness, nausea and vomiting, delirium, deep venous thrombosis and falls in-hospital. One falls in-hospital occurred in the FNB group, while none was observed in the IPB group. This fall might be attributed to quadriceps motor weakness caused by FNB. Although the incidence of falls in-hospital was low, we must remain vigilant about the potential adverse events due to quadriceps weakness.

There are several limitations in this study that should be considered when interpreting the results. First, the study population included patients undergoing hip arthroplasty due to a variety of conditions, including femoral neck fractures, femoral head necrosis, and hip osteoarthritis. The heterogeneity of this patient population could have introduced additional variability into the results, potentially reducing the likelihood of observing significant differences between groups. Future research may benefit from focusing on a more homogenous population, such as patients with hip fractures or those with non-inflammatory degenerative joint diseases like osteoarthritis.

Second, patients underwent both total hip arthroplasty and hip hemiarthroplasty were included in this study. This also adds heterogeneity and may have further reduced our ability to observe intergroup differences. Future studies might benefit from separately these surgical procedures. Third, patients in both the IPB and FNB groups reported relatively low pain levels postoperatively, suggesting that both techniques were effective for postoperative analgesia after hip arthroplasty. However, it would be suitable to add a control group with sham block to verify the effectiveness of IPB in the future study. Fourth, there was no blinding in this study when performing nerve blocks or analyzing data. This lack of blinding could have introduced bias in the outcome analysis. Lastly, the relatively small sample size may limit the robustness of some secondary outcomes. This size limitation also means we may have overlooked potential adverse effects of IPB, such as infection or in-hospital falls. Future studies with larger sample sizes could address these limitations and provide more reliable results.

## Conclusions

In summary, our study found no significant superiority of IPB over FNB in terms of postoperative analgesia following hip arthroplasty. However, IPB demonstrated notable advantages in terms of motor sparing, which may facilitate earlier recovery and rehabilitation for patients. Given these findings, IPB could be considered a viable alternative to FNB in the management of postoperative pain following hip arthroplasty. Further research is needed to optimize the application of IPB in clinical practice and explore its potential benefits in different patient populations.

## Data Availability

The data generated or analyzed during this study are not publicly accessible because they are part of ongoing research. However, the data are available from the corresponding author upon reasonable request.

## Abbreviations

FNB: Femoral nerve block
PENG block: Pericapsular nerve group block
IPB: Iliopsoas plane block
ASA: American Society of anesthesiologists
PACU: Post-anesthesia care unit
VAS: Visual analogue scale
MMT: Manual muscle testing
